# Development of omega-3 loxoprofen-loaded nanoemulsion to limit the side effect associated with NSAIDs in treatment of tooth pain

**DOI:** 10.1080/10717544.2021.1909179

**Published:** 2021-04-10

**Authors:** Khaled M. Hosny, Amal M. Sindi, Hala M. Alkhalidi, Mallesh Kurakula, Amira H. Hassan, Rana B. Bakhaidar, Walaa A. Abualsunun, Alshaimaa M. Almehmady, Ahmed Khames, Waleed Y. Rizg, Rasha A. Khallaf, Nabil K. Alruwaili, Nabil A. Alhakamy

**Affiliations:** a Department of Pharmaceutics, Faculty of Pharmacy, King Abdulaziz University, Jeddah, Saudi Arabia; b Center of Excellence for Drug Research and Pharmaceutical Industries, King Abdulaziz University, Jeddah, Saudi Arabia; c Oral Diagnostic Science Department, Faculty of Dentistry, King Abdulaziz University, Jeddah, Saudi Arabia; d Department of Clinical Pharmacy, Faculty of Pharmacy, King Abdulaziz University, Jeddah, Saudi Arabia; e Department of Biomedical Engineering, The Herff College of Engineering, Memphis, TN, USA; f Department of Pharmaceutics and Industrial Pharmacy, Faculty of Pharmacy, Beni-Suef University, Beni-Suef, Egypt; g Department of Pharmaceutics and Pharmacy Technology, College of Pharmacy, Taif University, Taif, Kingdom of Saudi Arabia; h Department of Pharmaceutics, Faculty of Pharmacy, Jouf University, Skaka, Saudi Arabia

**Keywords:** Loxoprofen, nanoemulsions, omega-3 oil, ulcer index, *ex vivo* permeation

## Abstract

The majority of newly developed drugs need to be incorporated with delivery systems to maximize their effect and minimize side effects. Nanoemulsions (NEs) are one type of delivery system that helps to improve the solubility and dissolution of drugs, attempting to enhance their bioavailability and onset of action. The objective of this investigation was to develop an omega-3 oil-based NE loaded with loxoprofen (LXP) to enhance its dissolution, *in vitro* release, and mucosal penetration and decrease its mucosal ulcerative effects when applied in an oral treatment. LXP-loaded NEs were formulated with varying levels of omega-3 oil (10–30%), surfactant polyoxyethylene-C21-ethers (laureth-21) (40–60%), and co-surfactant polyethylene glycol-40 hydrogenated castor oil (HCO-40) (30–50%) using an extreme vertices mixture design. The developed NEs were characterized for globule size and drug loading capacity. The optimal formulation was tested for *in vitro* drug release, *ex vivo* permeation, and ulcer index value. The developed NE acquired a globule size ranging 71–195 nm and drug loading capacity of 43–87%. Considering the results of the *in vitro* release study, the optimized NE formulation achieved 2.45-fold and 2-fold increases in drug permeation across tested mucosa compared to a marketed tablet and drug aqueous dispersion, respectively. Moreover, the optimum NE exhibited the best ulcer index in comparison to drug aqueous suspension and different formulations when tested in rats. Overall, this research highlights the capacity of NEs to deliver LXP with enhanced solubility, drug release, and permeation while effectively protecting the application site from side effects of the model drug.

## 1. Introduction

In recent years, several compounds with improved efficacy and therapeutic potential have been discovered. The use of such compounds has to be accompanied with the development of advanced drug delivery systems which are capable of conveying these active agents to the required sites with improved efficacy (Groo et al., 2017; Yousaf et al., 2019).

Non-steroidal anti-inflammatory drugs (NSAIDs) are a widely prescribed class of drugs for the management of inflammation, arthritis, and cardiovascular protection. However, they suffer from a variety of gastrointestinal disorders, such as ulcers and erosions, which could be mostly attributed to their cyclooxygenase (COX)-inhibiting effect that leads to a subsequent prostaglandin (PG) deficiency (Yousaf et al., 2019). The drop in PG secretion disables its cytoprotective effects and increases the susceptibility to mucosal erosions disorders, like gastric ulcers (Hirofumi et al., 2011; Groo et al., 2017).

Loxoprofen (LXP), an NSAID prodrug, contains the chemical structure of sodium-2-[4-(2-oxocyclopentyl-1-methyl) phenyl] propionate. It is considered as a potent analgesic for managing musculoskeletal and joint disorders in addition to pain and inflammation associated with several chronic and transient conditions (Tak et al., 2017). Following oral administration, LXP is absorbed from the gastrointestinal tract (GIT) as a free acid rather than as a sodium salt, which causes mild gastritis (Tyagi & Dhillon, 2012). LXP is swiftly metabolized in the liver by carbonyl reductase enzyme into its active trans-alcohol form, a potent and nonselective inhibitor of both isoforms of COX enzyme (i.e. COX-1 and COX-2) (Renton, 2011; Sarah & Garnock-Jones, 2016; Huda & Nidhal, 2019).

One of the most common uses of NSAIDs like LXP is to relieve pain and inflammation in teeth and buccal cavity, which is associated with dental disorders of hard and soft tissues of the teeth and the supporting bone. Moreover, patients could also suffer from some oral sores that might be due to bacterial or fungal infections. Although LXP has shown to be useful in such cases, its unwanted effect on the highly sensitive buccal mucosa may cause ulcerations. Therefore, it is recommended that drugs like LXP be accompanied by other agents to decrease their side effects.

Omega-3 fatty acids are long-chain polyunsaturated fatty acids composed of a mixture of α-linolenic (ALA, 18:3 n-3), eicosapentaenoic (EPA, 20:5 n-3), and docosahexaenoic (DHA, 22:6 n-3) acids. Vegetable oils, such as olive and flaxseed oil, are rich in ALA while, marine oils are major sources of EPA and DHA (Zhao et al., 2019). Since the human body is unable to synthesize omega-3 fatty acids, which are important to our diet, they must be supplied from external sources (Lavie et al., 2009). There is increasing evidence of the role of omega-3 fatty acids in inflammation management and immune response regulation. These fatty acids exert such effects through reducing the production of cytokine inflammatory mediators, like interleukin-1 and 6 (IL-1, IL-6) and tumor necrosis factor-α (TNF-α) (Calder, 2013). Several interlinked mechanisms explain the anti-inflammatory effects of omega-3 fatty acids, including the alteration of cell membrane phospholipids, lipid raft disruption, inhibition of pro-inflammatory transcription factor nuclear factor kappa B, decreased inflammatory genes expression, and the activation of anti-inflammatory transcription factor NR1C3, which attaches to the G protein coupled receptor (i.e. GPR120). Several studies have reported the use of omega-3 in the treatment of different forms of nephritides in adult humans (Mayer et al., 2003; Yamada et al., 2008; Beck et al., 2013). Moreover, many animal studies reveal the ability of omega-3 fatty acids to reduce the production of inflammatory mediators in rheumatoid arthritis and inflammatory bowel diseases (Ferrucci et al., 2006; Patel et al., 2013; Yuzawa et al., 2016). Therefore, omega-3 fatty acids appear to be good candidates for incorporating with LXP to reduce inflammation and ulcerative effects associated with the drug (Hosny et al., 2019).

Nanoemulsions (NEs) are thermodynamically stable dispersions of oil and water that are stabilized by an interfacial film of surface active agents (Nanthakumar et al., 2016; Stoyanova et al., 2016; Anuar et al., 2020). They usually are prepared in a droplet size ranging from 100 to 1000 nm. Routinely, NEs can be fabricated in the form of oil-in-water (O/W), water-in-oil (W/O), and multiple emulsions (W/O/W) (Abdelbary et al., 2017). NE formulations offer several advantages compared to conventional emulsions, including: (1) the ability to incorporate different hydrophilic and hydrophobic drugs; (2) localization of therapeutic agents at the required sites and decreased side effects due to their very large surface area supplied by the very small size of their droplets; (3) improved drug solubility and bioavailability; and (4) ability to achieve sustained drug release (Ferrucci et al., 2006; Abdelbary et al., 2017).

A toothache or tooth pain is caused when the nerve in the root of a tooth or surrounding a tooth is irritated. Dental (tooth) infection, decay, injury, or loss of a tooth are the most common causes of dental pain (Shankland, 2001). Pain may also occur after an extraction (tooth is pulled out). Pain sometimes originates from other areas and radiates to the jaw, thus appearing to be tooth pain (Gremillion, 2002)

Accordingly, the objective of the present study was to formulate omega-3 fatty acid-based NEs for improving LXP solubility in buccal media to provide faster onset of action and reduce the ulcerative side effects that might be associated with its use for the treatment of oral pain and inflammation.

## 2. Materials and methods

### 2.1. Materials

Loxoprofen was procured from Sigma-Aldrich (St. Louis, MO). Omega-3 oil was obtained from Acros Organics (Geel, Belgium; Morris Plains, NJ). Propylene glycol and cremophor EL were purchased from Spectrum Chemical Manufacturing Corporation (Gardena, CA). Polyethylene glycol-25-Stearate (MYS-25V), polyethylene glycol-40, -50, and -60 hydrogenated castor oil (HCO-40, HCO-50, and HCO-60), polyoxyethylene-C21-ethers (laureth-21), polyoxyethylene lanoline (Sorbeth-20), and sefsol were obtained from Nikko Chemicals Co., Ltd. (Tokyo, Japan). All other chemicals and reagents were of analytical grade.

### 2.2. Methodology

#### 2.2.1. Solubility studies

LXP solubility was assessed in different surfactants and co-surfactants to obtain variable self-emulsifying regions that could help design the omega-3 oil based NE formulations loaded with LXP. Excess amounts of LXP were dissolved in 5 mL of various tested surfactants (MYS-25V, laureth-21, Sorbeth-20, and Cremophor EL) and co-surfactants (HCO-40, HCO-50, HCO-60, and propylene glycol) to define drug solubility in each individual solution. Vials containing the tested solutions were stored for 72 h at 25 ± 2 °C in a water bath (model 1031; GFL Corporation, Hamburg, Germany). After equilibrium was obtained, the prepared solutions were centrifuged to remove excess drug (Sigma 3k30, Osterode am Harz, Germany) at 4500 rpm for a period of 15 min. The supernatants were removed, diluted with methanol, and assessed for their LXP content via high-performance liquid chromatography (HPLC) method with Phenomenex, Luna C18(2) (250 × 4.6 mm, 5 μ) column. In this procedure, a mixture of 0.4% orthophosphoric acid and acetonitrile in a ratio of 45:55 (v/v) was used as the mobile phase with a flow rate of 1.0 mL/min. UV detection of LXP was performed at 210 nm (Patel et al., 2013).

#### 2.2.2. Tenacity of loxoprofen-omega-3 oil emulsifying regions in selected surfactants and co-surfactants

The self-emulsifying regions were located using pseudo-ternary phase diagrams in order to estimate the lower and upper levels for each component of the self-nanoemulsified drug delivery systems (SNEDDs), which were selected according to the solubility study. Within these levels, mixtures of drug, oil, the selected surfactant, and co-surfactant impulsively developed a translucent emulsion with nano-size droplets when diluted with water under mild stirring conditions. Therefore, these diagrams were considered to be a useful method to assess the required levels for each SNEDDs component. The resulting mixture was examined based on the globule size uniformity and distribution using a Zetatrac Instrument (Microtrac Inc., Montgomeryville, PA). Determination of the emulsion region in the phase region could be identified as NE region when droplet size is higher than 1 nm and less than 1 μm. Then, the NE formulations were loaded with LXP and evaluated.

#### 2.2.3. Experimental design and optimization of loxoprofen-omega-3 oil self nanoemulsion formulations (LXP-O3-SNEDDs)

The experimental design was employed for the development and optimization of LXP-O3-SNEDDS due to its high efficiency in evaluation of the prepared formulations and prediction of optimum solutions. A mixture design based on extreme vertices was applied to determine the effects of the independent variables (i.e. omega-3 oil (A), laureth-21 (B), and HCO-40 (C) in 17 formulations). The dependent responses included the globule size of the prepared SNEDDS (*Y*_1_) and drug loading capacity (*Y*_2_). Response variables were recorded along with the optimization of the formulation using DOE. Different components and their ratios along with the measured responses and their constrains that were selected for optimization using DOE are depicted in Table 1.

**Table 1.** Levels of independent variables along with dependent variables and their constraints.

Independent variables	Levels	
	Low	High
A: Omega-3 oil %	10.0	30.0
B: laureth-21 %	40.0	60.0
C: HCO-40 %	30.0	50.0
Dependent variables	Constrains	
Globule size (*Y*_1_)	Minimize	
Drug loading capacity (*Y*_2_)	Maximize	

#### 2.2.4. LXP-O3-SNEDDs visual inspection

The efficiency of LXP-O3-SNEDDs formation was visually observed for clarity and signs of instability, such as coagulation or cracking of spontaneously formed NEs.

#### 2.2.5. Droplet size measurement (Y_1_)

Two hundred microliters of each formulation was diluted with 800 μL double distillate deionized water and vortexed for 5 min. Aliquots of 200 μL were withdrawn from the diluted samples for droplet size determination using a dynamic light scattering technique with a Zetatrac instrument (Microtrac Inc., Montgomeryville, PA).

#### 2.2.6. Evaluation of LXP-O3-SNEDDs drug loading capacity (Y_2_)

Loxoprofen loading was determined in each SNEDDs mixture by dissolving a known excess amount of LXP in 1 g plain SNEDDs separately. The mixtures were filled into vials and stored at 25 ± 2 °C for 24 h in a shaking water bath. After obtaining equilibrium, the prepared mixtures were centrifuged for about 15 min at 4500 rpm. The precipitates were collected, washed, and dispersed in methanol. Then, the drug was extracted, and the amount of LXP was determined using the previously mentioned HPLC method in which, the mobile phase was methanol–water–acetic acid–triethylamine(600:400:1:1). The detection wavelength was set at 222 nm. The flow rate was 1.00 mL·min^–1^. The column temperature was 25 °C. The drug loading capacity was determined using the following equation:

Drug loading capacity=drug content in the product obtained (mg)/total product weight (mg)×100.

#### 2.2.7. Optimization of LXP-O3-SNEDDs

Parameters, such as F-ratio, *p* value, and degrees of freedom, for all independent variables and their interactions were determined for analysis of variance (ANOVA) of the obtained models. Based on the results, the responses were used to identify the model that best fit the obtained data. Specifically, *p* values lower than .05 indicated that the investigated model terms were significant. In addition, CV% values, determination coefficients, predicted (*R*^2^), and adjusted (*R*^2^) were employed to assess the model fitness. The optimum variables were selected on the basis that the prepared LXP-O3-SNEDDs could achieve the minimum globule and maximum drug loading. Furthermore, about 50 mL of the optimized formula was prepared and evaluated for the *in vitro* release, *ex vivo* permeation, and ulcer index.

#### 2.2.8. Characterization of optimized LXP-O3-SNEDDs

The optimized formulation was developed and evaluated by measuring its globule size, and drug loading. Following that, it was subjected to an *ex vivo* permeation study, *in vitro* release study, and ulcer index determination.

#### 2.2.9. *In vitro* release of loxoprofen from different NE formulations

USP dissolution tester (apparatus I) was employed in this investigation. Glass cylindrical tubes with a 2.7 cm diameter and 10 cm length, containing the tested formulations, were attached to the rotating shafts instead of baskets and tightly covered with semipermeable membranes (100-µm pore size). The tested formulations (1 g each of optimized LXP-O3-SNEDDs containing 60 mg LXP, a commercially available 60 mg LXP tablet, and LXP aqueous suspension) were inserted into the glass tubes. Then, the tubes were placed in a 50 mL of phosphate buffer saline (pH 6.8). The release study was carried out at 37 ± 0.5 °C, and the medium was stirred at 25 rpm. Samples were withdrawn from the dissolution medium at different time intervals for 15 min. The amount of released LXP was quantified by measuring the absorbance via the previously mentioned HPLC method.

#### 2.2.10. *Ex vivo* skin permeation study of optimized LXP-O3-SNEDDs

*Ex vivo* studies were carried out for the optimized LXP-O3-SNEDDs, commercially LXP tablet, and LXP aqueous suspension, each containing 60 mg LXP. Sheep buccal mucosa obtained from a local slaughterhouse was used as a model permeation membrane, and an automated Franz diffusion cell (MicroettePlus^®^, Hanson Research, Chatsworth, CA) was employed as the apparatus. The prepared sheep buccal mucosa (2 × 2 cm) was carefully mounted between donor and receptor compartments of the Franz diffusion cell (1.75 cm^2^). The receptor chamber contained 8 mL PBS (pH 6.8), temperature was maintained at 32 ± 2 °C, and the media was stirred at a speed between 410 and 430 rpm. Definite aliquots were auto sampled at predetermined intervals and quantified for LXP content using the HPLC method. Drug diffused across the mounted mucosa was elucidated by plotting the cumulative amount of permeated LXP (Q) per unit area against time. Important parameters, such as *J*_ss_ (steady-state flux), Pc (permeability coefficient), EF (enhancement factor), and *D* (diffusion coefficient), were calculated from the obtained diffusion data. The comparative diffusion patterns with respect to various formulations were plotted. The percentage of permeated LXP and total amount of LXP diffused across the receptor chamber were calculated by the following equation (Shankland, 2001):

(1) $$\text{Percentage}\;\text{permeated}\;=\;\left[L_{p}/L_{T}\right]\;\times \;100$$

where *L*_p_ is the amount of LXP that permeated into the receptor chamber and *L*_T_ is the initial amount of LXP in the donor chamber.

#### 2.2.11. Ulcer index determination

Male albino rats weighing between 150 and 250 g selected for this study were procured from the animal house facility of the Clinical Laboratory Center, Beni-Suef, Egypt. The present study was approved by the Institutional Review Board for Animal Research/Studies Animals (approval no. 11-01-21). Five animal groups (three animals per group) were tested to determine the ulcer index according to the previously reported method (Sindi & Hosny, 2019), as follows. Group 1 received normal saline only and served as the negative control; group 2 was treated with an aqueous dispersion of LXP and served as the positive control; group 3 was treated with the LXP-omega-3 dispersion; group 4 was treated with optimized LXP-SNEDDs prepared without omega-3; and group 5 was treated with optimized LXP-O3- SNEDDs. The ulcerative effect on mouth mucosa in each group was scored from 1 to 5 after three days of treatment with each formulation; the scoring system was assigned as follows: 1 (normal-colored epithelial lining), 2 (red coloration), 3 (spot ulceration less than 1 mm), 4 (ulcers >1 mm but without hemorrhagic streaks), 5 (ulcers >1 mm and with hemorrhagic streaks), and the results were noted and recorded.

## 3. Results and discussion

### 3.1. Solubility studies of LXP

**Figure 1.**
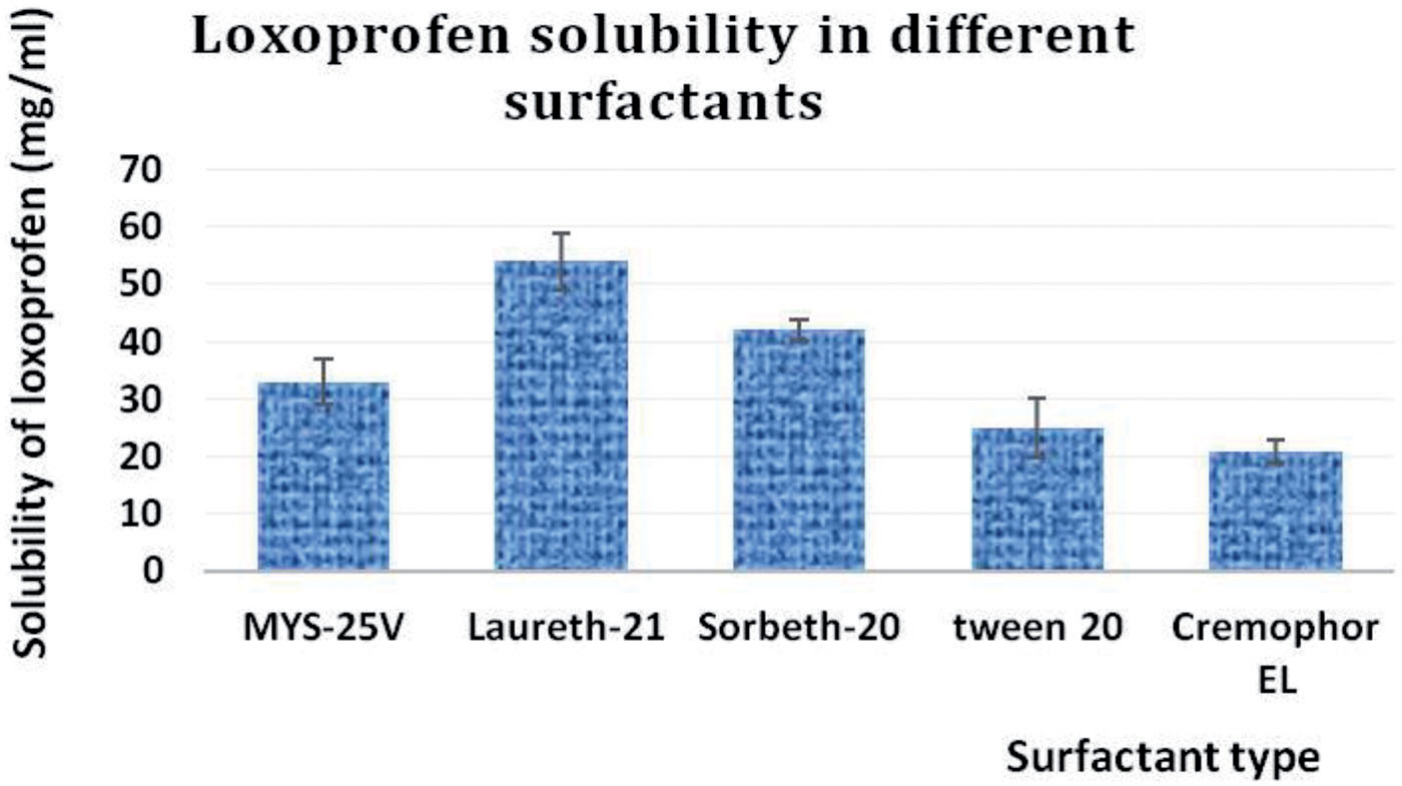
Solubility of LXP in different surfactants.

**Figure 2.**
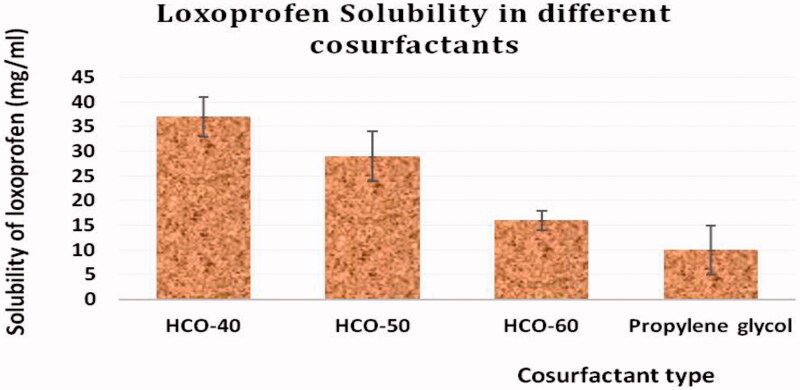
The solubility of LXP in different co-surfactants.

The results of LXP solubility studies in different surfactants and co-surfactants revealed that the tested therapeutic agent had the highest solubility of the surfactant, laureth-21, and the co-surfactant, HCO-40. Figures 1 and 2 illustrate the relation between the percent of solubilized LXP and the type of the used surfactants and co-surfactants. Accordingly, laureth-21 and HCO-40 were selected for constructing the pseudo-ternary phase diagram in order to estimate the lower and upper levels of each component of SNEDDs.

### 3.2. Tenacity of loxoprofen-omega-3 oil emulsifying regions in selected surfactants and co-surfactants

The emulsification process was performed by mixing water and oil and stabilizing the formed system using a surfactant (Sindi & Hosny, 2019) favoring particle size reduction (Yamada et al., 2008). The oil phase is usually composed of non-polar and hydrophobic substances consisting of hydrocarbons, natural triglycerides, or other molecules (Tsai et al., 2014), while the aqueous phase usually contains solutes and electrolytes solubilized in water. In the current research, the pseudo-ternary phase diagram was constructed to determine the accurate concentration range of the oil, surfactant, and co-surfactant that would yield regions of NE.

**Figure 3.**
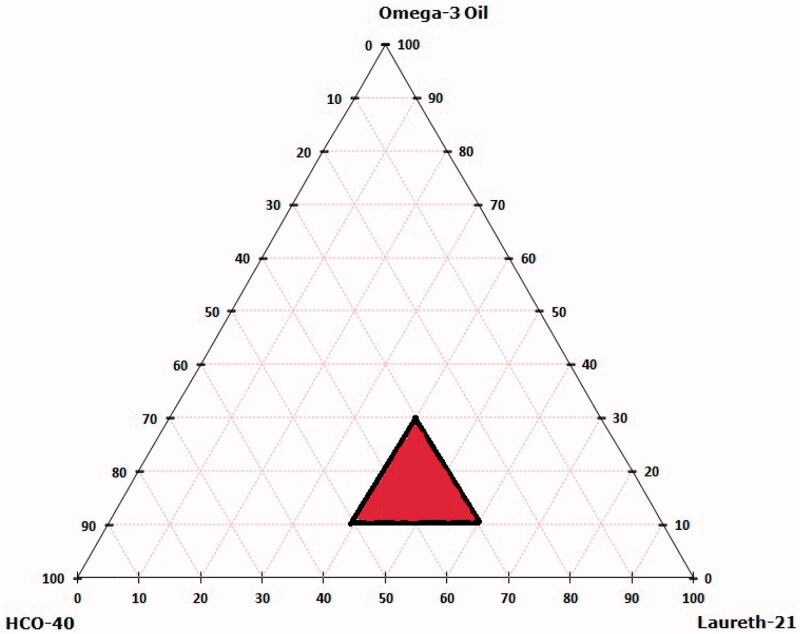
The pseudo-ternary phase diagram of omega-3 oil, laureth-21 surfactant, and HCO-40 co-surfactant.

As could be seen in the pseudo-ternary phase diagram in Figure 3, the precise concentration ranges of omega-3 oil, laureth-21, and HCO-40 for preparing NEs were determined to be 10–30, 40–60, and 30–50%, respectively. Therefore, these concentration ranges were applied to develop the experimental design that was subsequently employed for the production and optimization of different LXP-O3-SNEDDs formulations.

### 3.3. Visual inspection of LXP-O3-SNEDDs

The visual inspection of LXP-O3-SNEDDs formulations showed clear translucent dispersions with no signs of coagulation or cracking. This suggests that the determined concentration ranges were precise in the formation of stable NEs.

### 3.4. Droplet size measurement (*Y*_1_)

The droplet size of the developed NE was in the range of 71–195 nm (Table 2) with PDI values from 0.1 to 0.3, which reveal the acceptable stability, homogeneity, and size distribution of the developed formulations.

**Table 2.** Extreme vertices mixture design responses of LXP-O3-SNEDDs.

	A	B	C	*Y*_1_	*Y*_2_	
	Omega-3 oil %	Laureth-21 %	HCO-40 %	Globule size (nm)	Loading capacity (%)	PDI
1	16.6667	40	43.3333	151	47	0.12
2	30	40	30	195	57	0.21
3	13.3333	53.3333	33.3333	110	82	0.10
4	16.6667	46.6667	36.6667	142	70	0.25
5	10	46.6667	43.3333	83	66	0.30
6	10	60	30	72	87	0.29
7	10	53.3333	36.6667	77	80	0.26
8	23.3333	43.3333	33.3333	170	63	0.27
9	10	60	30	71	86	0.30
10	0.3	40	30	194	56	0.16
11	16.6667	46.6667	36.6667	141	71	0.13
12	16.6667	53.3333	30	132	83	0.10
13	10	40	30	94	44	0.15
14	23.3333	46.6667	30	162	74	0.20
15	23.3333	40	36.6667	178	50	0.22
16	10	40	50	92	43	0.26
17	13.3333	43.3333	43.3333	120	60	0.19

A cubic model of polynomial analysis was then applied on the obtained globule size data. The used experimental design revealed the studied model's efficiency to assess the significant effect of omega-3 oil (A), laureth-21 (B), and HCO-40 (C) on the globule size of LXP-O3-SNEDDs. The selected model exhibited an adjusted *R*^2^ value of 0.9994 and expected *R*^2^ of 0.9983, which are in close accordance, as presented in Table 3. Data analysis by ANOVA resulted in the following equation:

Globule size= +194.43A+71.45B+92.96C +62.94 AB+92.91 AC−11.13BC+138.56ABC  −71.24AB(A−B)−40.85AC(A−C)+10.14BC(B−C) 

**Table 3.** Regression analysis results for *Y*_1_ and *Y*_2_ responses.

Dependent variables	*R* ^2^	Adjusted *R*^2^	Predicted *R*^2^	*p* Value	*F*-value	Adequate precision
*Y*_1_	0.9997	0.9994	0.9983	.0001	2794.69	149.2959
*Y*_2_	0.9990	0.9980	0.9975	.0001	986.80	87.7932

Based on the above equation, all of the studied factors significantly affect globule size at a *p* value <.0001. Nevertheless, omega-3 oil exhibited the most dominant effect on globule size with the highest coefficient of 194.43 compared to laureth-21 (71.45) and HCO-40 (92.96). In addition, the interaction terms involving parameter (*A*), such as (*AB*) and (*AC*), attained higher coefficient values (i.e. 62.94 and 92.91, respectively) compared to the interaction term (*BC*) that does not involve factor (*A*), which exhibited a coefficient value of 11.13. The increase in droplet size relative to increased omega-3 oil presence could be explained by the fact that increased oil % decreases the surfactant and co-surfactant percentage and, thus, reduces their ability to reduce the droplet size. As a result, larger oil globules are obtained, which is similar to results reported in literature (Kumar et al., 2019; Sindi & Hosny, 2019).

**Figure 4.**
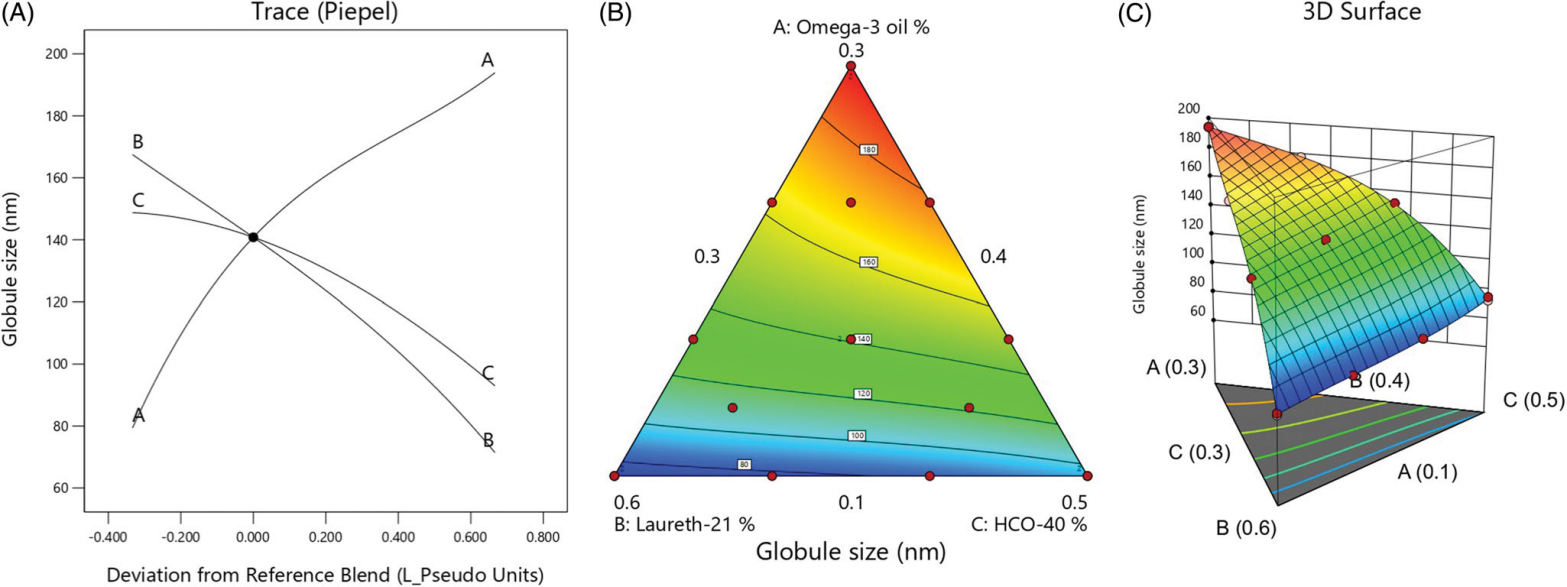
(A) Main  effect diagram, (B) contour plot, and (C) 3D surface plot showing the effects of different independent variables on the globule size of different LXP-O3-SNEDDs formulations.

Figure 4 presents the main effect diagram, contour plot, and 3D-surface plots that reveal the effect of the factors on LXP-O3-SNEDDs droplet size. These graphs confirm that the globule size of the developed NEs was dependent on the presence of omega-3 in the formulations.

According to Figure 4(A), it is apparent that the increase in surfactant and co-surfactant percentages decreased the globule size due to their ability to reduce interfacial tension between aqueous and organics phases and, thus, produce smaller droplets.

### 3.5. Evaluation of LXP-O3-SNEDDs drug loading capacity (*Y*_2_)

The loading of LXP in the developed NE formulations was found to be between 43 and 87%, as shown in Table 2.

A special quartic model of polynomial analysis was applied to the obtained drug loading data. The used experimental design revealed the model's efficiency to assess the significant effects of omega-3 oil (A), laureth-21 (B), and HCO-40 (C) on the LXP-O3-SNEDDs drug loading capacity. The selected model attained an adjusted *R*^2^ value of 0.9980 and expected *R*^2^ of 0.9975, as presented in Table 3. Data analysis by ANOVA resulted in the following equation:

$$\text{Loading}\;\text{capacity}=\;+56.49\;A\;+86.41B+43.64C\;+31.91AB-6.86AC\;+36.06BC\;-81.73A^{2}BC-31.89\;AB^{2}C+250.58ABC²$$

As could be seen from the above equation, all the studied factors had a significant effect on drug loading at a *p* value <.0001.

However, the parameter with the most dominant effect on drug loading was the surfactant, as it attained the highest coefficient (86.41) when compared to omega-3 oil (56.49) and HCO-40 (43.64). In addition, the interaction terms involving parameter (*B*), such as (*AB*) and (*BC*), attained higher coefficient values (31.92 and 36.06, respectively) compared to the interaction term (*BC*) that does not involve factor (*A*), which exhibited a coefficient value of 6.86.

**Figure 5.**
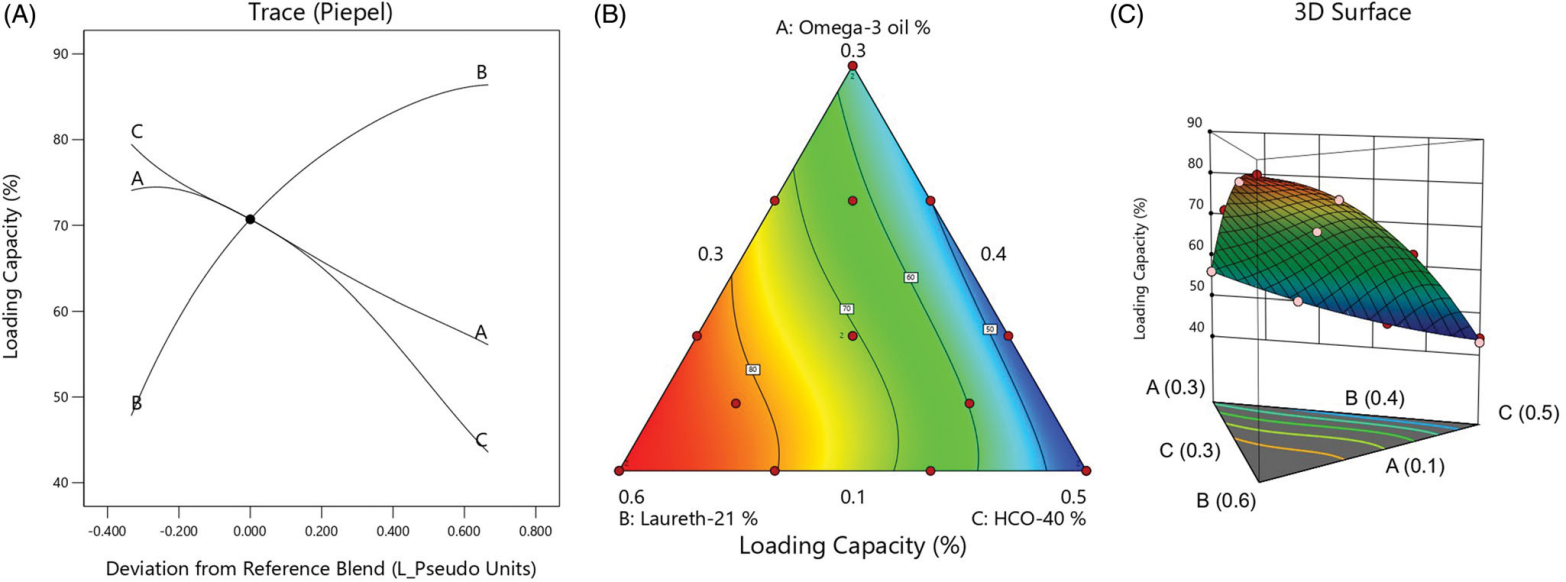
(A) Main effect diagram, (B) contour plot, and (C) 3D surface plot showing the effects of different independent variables on the drug loading of different LXP-O3-SNEDDs formulations.

Moreover, Figure 5 presents the main effect diagram, contour plot, and 3D-surface plots that reveal the effect of the studied factors on LXP-O3-SNEDDs drug loading.

According to Figure 5(A), it could be noticed that the increase in surfactant would increase the drug loading %. Such effect might be due to its ability to increase the accommodation of the hydrophilic drug in the developed NE and, hence, increase the drug loading %.

### 3.6. Optimization of LXP-O3-SNEDDs

**Figure 6.**
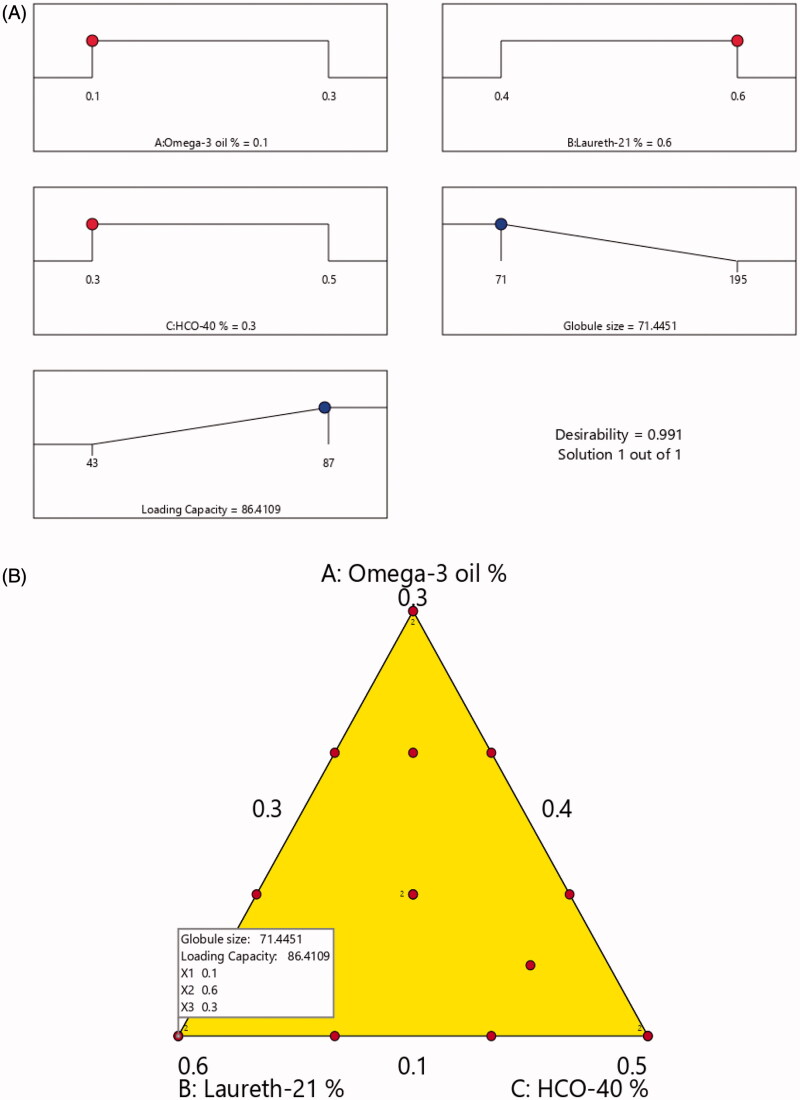
(A) Desirability ramp revealing the levels of independent variables and predicted values for the responses of the optimum formulation and (B) overlay plot for determining the optimal LXP-O3-SNEDDs region.

From the above data, the optimized NE formulation was produced using the most suitable characteristics. The Stat graphics software proposed several solutions representing various combinations of the studied factors, specifically the optimum formulation consisted of 10% omega-3 oil, 60% laureth-21, and 30% HCO-40. The developed optimized formulation attained a globule size of 71.44 nm and drug loading of 86.41% with 0.991 desirability. Figure 6 presents the desirability ramp that reveals the levels of the independent variables and predicted values of the measured responses of the optimum formulation. Table 4 proves that the actual and expected values of the optimum formulation’s parameters were in a very close accordance with no significant differences (*p*>.05), which confirms the equations’ precision and validity.

**Table 4.** Actual and experimental values of the optimized nanoemulsion formulation.

Solution	Omega-3 oil %	Laureth-21 %	HCO-40 %	Droplet size (nm)	Drug loading (%)	Desirability
Predicated value	10	60	30	71.44	86.40	0.991
Experimental value	10	60	30	73	85	0.991

### 3.7. Check point analysis

Adjusted and predicted *R*^2^ values of the measured responses were also closely related, suggesting the significance of the design’s predicting capacity. According to Table 5, actual/expected ratios with low percentage error and good residuals were observed between the experimental and predicated dependent variables, as proven by the lack of curvature in the responses. Figure 6 illustrates the desirability ramp and overlay plot for the optimal region.

**Table 5.** Composition, actual, and predicted responses of the optimal nanoemulsion formulation.

Factor	Optimal value	Response variable	Actual value	Predicted value	% Prediction error^a^
A: omega-3 oil %	10	Droplet size (nm)	73	71.44	0.021
B: laureth-21 %	60	Drug loading (%)	85	86.4	–0.016
C: HCO-40 %	30				

^a^ Calculated as (actual – predicted/actual)×100.

### 3.8. *In vitro* release of loxoprofen from different NE formulations

**Figure 7.**
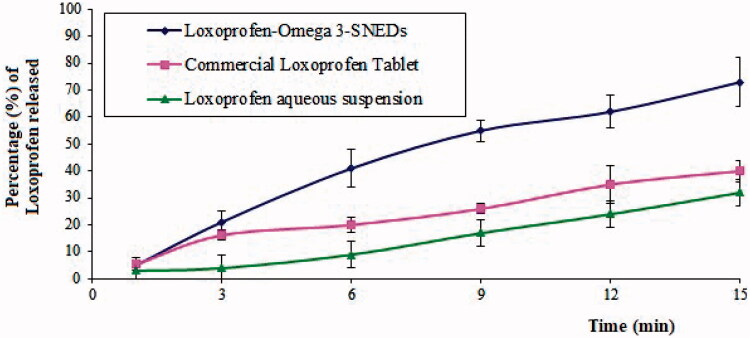
The *in vitro* release profiles of LXP from tested formulations.

The drug release profiles of the tested formulations in Figure 7 show that LXP-O3-SNEDDs attained the highest cumulative percentage of the drug released (73% after 15 min) compared to 40% and 32% of the marketed tablet and aqueous suspension of LXP, respectively.

Such improvement in drug release in the optimized formulation compared to the other tested formulas might be due to the NE content of the surfactant and co-surfactant, which could increase drug dissolution and, hence, drug release. Moreover, the small globule size of NE offers a larger surface area for drug release and helps to increase the amount of drug release, allowing for better absorbance and faster onset of action.

### 3.9. *Ex vivo* skin permeation study of the optimized LXP-O3-SNEDDs

The cumulative amounts of LXP permeated after 1 h from the optimized LXP-O3-SNEDDs, marketed LXP tablet, and aqueous LXP suspension were 8823, 3600, and 4000 μg/cm^2^, respectively. The *J*_ss_, *D*, and *P* of optimized LXP-O3-SNEDDs were greater in comparison to a marketed tablet and aqueous suspension with respect to the other examined formulations. As illustrated in Table 6, the optimized LXP-O3-SNEDDs achieved maximum LXP permeation across the oral mucosa.

**Table 6.** Permeation parameters of loxoprofen across buccal mucosa for different formulations.

Parameters of permeation	Optimized LXP-O3-SNEDDs formulation	Loxoprofen marketed tablet	Loxoprofen aqueous suspension
Cumulative amount permeated (μg/cm^2^)	8823	3600	4000
Steady state flux, *J*_ss_, (μg/cm^2^.min)	145.2	60.4	67.8
Permeability coefficient, *P*, (cm/min)	12.1 × 10^–3^	4.6 × 10^–3^	5.0 × 10^–3^
Diffusion coefficient, *D*, (cm^2^/min)	6.32 × 10^–4^	2.98 × 10^–4^	3.13 × 10^–4^
Relative permeation rate (RPR)^a^	2.45	–	1.11
Enhancement factor (*F*_en_)^b^	2.20	0.9	–

^a^ Relative permeation rate (RPR)=cumulative amount permeated_Test_/cumulative amount permeated_Marketed_.

^b^ Enhancement factor (*F*_en_)=cumulative amount permeated_Test_/cumulative amount permeated_aqueous dispersion_.

The increase in drug permeation in the case of the optimized formulation could be due to its surfactant content, which could act as a permeation enhancer in addition to the nano-sized droplets of NE. This could provide a broad surface area and allow more drug to penetrate across the tested mucosa.

### 3.10. Ulcer index determination

As observed in Table 7, the ulcer index values of different rat groups are quite diverse. The animal group that received pure LXP had the greatest ulcer index, while those that received the optimized LXP-O3-SNEDDs had the lowest ulcer index equal to that of the negative control group, which received only normal saline. It was also noticed that the rats which received dispersion of LXP and omega-3 oil had lower ulcer index than those that received optimized LXP SNEDDs prepared without omega-3.

**Table 7.** Ulcer index values of different rat groups.

Tested formulations	Ulcer index
Normal saline	1
Aqueous dispersion of loxoprofen	5
Loxoprofen-omega-3 dispersion	2
Optimized loxoprofen SNEDDs prepared without omega-3 oil	3
Optimized loxoprofen-omega3 SNEDDs	1

From the above data, it could be concluded that the presence of omega-3 oil can greatly decrease the ulcer causing characteristics of LXP. Such effect of omega-3 oil could be ascribed to its capacity to reduce the production of cytokine inflammatory mediators via hindering pro-inflammatory transcription factor nuclear factor kappa B with the following decrease in inflammatory genes expression, moreover, it might activate the anti-inflammatory transcription factor NR1C3 and bind tightly to the G protein coupled receptor (i.e. GPR120).

Collectively, omega-3 oil could be considered a good protecting substance that can be safely incorporated with NSAIDs such as LXP to reduce its unwanted eroding effects on the highly sensitive buccal mucosa during treatment of pain and inflammation associated with buccal or dental conditions.

## 4. Conclusions

The obtained NEs showed a good ability to improve the dissolution and permeation of LXP through buccal mucosa. The pseudo-ternary phase diagram was successfully developed to determine the optimum concentrations of omega-3 oil, laureth-21, and HCO-40 mixture and subsequently assign the NE region satisfactory for preparing the drug delivery system. The developed NE acquired a globule size from 71 to 195 nm with adequate homogeneity and drug loading capacity of 43–87%. In the *in vitro* release study, the optimized NE formulation revealed the highest drug release and 2.45-fold and 2-fold increases in drug permeation across tested mucosa compared to the marketed tablet and drug aqueous dispersion, respectively. When tested in rats, the optimum NE achieved the best ulcer index in comparison to different formulations. In brief, this research demonstrates that omega-3-based LXP-loaded NEs can provide better drug dissolution, permeation, and, hence, faster onset of drug action, while effectively inhibiting the ulcerative side effects of the drug and improving patient adherence.
